# Small RNA-seq analysis of single porcine blastocysts revealed that maternal estradiol-17beta exposure does not affect miRNA isoform (isomiR) expression

**DOI:** 10.1186/s12864-018-4954-9

**Published:** 2018-08-06

**Authors:** Jochen T. Bick, Veronika L. Flöter, Mark D. Robinson, Stefan Bauersachs, Susanne E. Ulbrich

**Affiliations:** 1 ETH Zurich, Animal Physiology, Institute of Agricultural Sciences, Zurich, Switzerland; 2 Physiology Weihenstephan, Technische Universität München, Freising, Germany; 3 Institute of Molecular Life Sciences and SIB Swiss Institute of Bioinformatics, University of Zurich, Zurich, Switzerland; 4 University of Zurich, Genetics and Functional Genomics, Clinic for Animal Reproduction Medicine, Zurich, Switzerland

**Keywords:** miRNAs, isomiRs, Small RNA-seq, Pig embryos, Estradiol treatment

## Abstract

**Background:**

The expression of microRNAs (miRNAs) is essential for the proper development of the mammalian embryo. A maternal exposure to endocrine disrupting chemicals during preimplantation bears the potential for transgenerational inheritance of disease through the epigenetic perturbation of the developing embryo. A comprehensive assembly of embryo-specific miRNAs and respective isoforms (isomiR) is lacking to date. We aimed at revealing the sex-specific miRNA expression profile of single porcine blastocysts developing in gilts orally exposed to exogenous estradiol-17 (E2). Therefore we analyzed the miRNA profile specifically focusing on isomiRs and potentially embryo-specific miRNAs.

**Results:**

Deep sequencing of small RNA (small RNA-seq) result in the detection of miRNA sequences mapping to known and predicted porcine miRNAs as well as novel miRNAs highly conserved in human and cattle. A set of highly abundant miRNAs and a large number of rarely expressed miRNAs were identified by using a small RNA analysis pipeline, which was integrated into a novel Galaxy workflow specifically benefits incompletely annotated species. In particular, orthologue species information increased the total number of annotated miRNAs, while mapping to other non-coding RNAs avoided falsely annotated miRNAs. Neither the low nor the high dose of E2 treatment (10 and 1000 µ E2/kg body weight daily, respectively) affected the miRNA profile in blastocysts despite the distinct differential mRNA expression and DNA methylation found in previous studies. The high number of generated sequence reads enabled a comprehensive analysis of the isomiR repertoire showing various templated and non-templated modifications. Furthermore, potentially blastocyst-specific miRNAs were identified.

**Conclusions:**

In pre-implantation embryos, numerous distinct isomiRs were discovered indicating a high complexity of miRNA expression. Neither the sex of the embryo nor a maternal E2 exposure affected the miRNA expression profile of developing porcine blastocysts. The adaptation to the continuous duration of the E2 treatment might explain the lack of an effect.

**Electronic supplementary material:**

The online version of this article (10.1186/s12864-018-4954-9) contains supplementary material, which is available to authorized users.

## Background

Endocrine disrupting chemicals (EDCs) are natural and synthetic substances that may impact on health by deteriorating the endogenous hormone system [1, 2]. During the preimplantation phase the embryo is specifically sensitive towards EDC acting as transcriptional modulators [3, 4]. EDC may perturb the uterine milieu and thereby also indirectly impose adverse transgenerational effects on the developing embryo [5]. Depending on the time, dose and EDC substance, the effects may be either detrimental or only lead to minor effects in the targeted individual or the subsequent offspring. Estradiol-17 (E2) is the most potent endogenous estrogen in the body. Because it is present in the environment and can adversely act on the developing organisms, it is considered as an EDC [6, 7].

In pigs, embryo development is under maternal control until the 4-cell stage. While embryonic genome activation occurs at around 3 days post fertilization [8], both compact morula and the subsequent blastocyst are formed in the uterus at around 4–5 days after fertilization, respectively [8]. At Day 7 of development, the embryo hatches from the zona pellucida, increases in size until Day 10 of development [9] and starts to rapidly elongate by Day 11. At the same time, the embryo secretes E2 as primary pregnancy recognition signal [10].

We have only recently shown that gestational low-dose E2 treatment in pigs altered body composition and bone development in male and female offspring, respectively [6, 11]. Specifically, the oral application of very low doses of E2 left a lasting epigenetic fingerprint in both the mother and her offspring, revealed by differential gene expression and DNA methylation of cell cycle regulation and tumor suppressor genes in a number of different tissues [12]. An intergenerational effect of gestational exposure was revealed as blastocysts not only showed differential gene expression, but also differential local DNA methylation patterns largely similar to the maternal tissues [12] (Flöter et al, under review). In embryos of several species including mice, cattle, and pigs, a dynamic developmental stage-specific microRNA (miRNA) expression has been described [13–15]. In addition, it has been hypothesized that embryonic E2 in pigs is also associated with changes in the expression of miRNAs in the embryo [13], which can lead to pregnancy disruption [16].

In this study we focus on non-coding RNA (ncRNA) which are transcripts that are not translated into proteins and usually have structural or regulatory roles [17]. The corresponding genes are located in intergenic and intronic regions or on the reverse strand of protein-coding genes, but can also have similar structures as protein-coding genes located within protein-coding sequences [18]. Many known ncRNAs are assigned to different functional classes [19] and play a pivotal role in gene expression regulation. Well-known long ncRNAs present in very high amounts in the cell are rRNAs and tRNAs. Other small ncRNA groups, such as long piRNAs and miRNAs with sizes below 50 nt, have only lately been studied intensively [20]. Due to the small size, the latter had been difficult to discriminate from partially degraded longer transcripts [21]. While piRNAs have been shown to mediate transposon silencing and epigenetic gene regulation in gametes [22], miRNAs comprise a large class of small ncRNAs involved in post-transcriptional repression of genes, inhibition of messenger RNA (mRNA) translation and/or enhanced mRNA degradation [23, 24]. MiRNAs are produced from a stem-loop-containing primary precursor (pri-miRNA) [25] that is processed by the Drosha-DGCR8 complex into a precursor hairpin (pre-miRNA) of ∼70nt length in the nucleus. The pre-miRNA is transported into the cytoplasm where it is converted by Dicer to a 17–27 nucleotides long double stranded miRNA/miRNA* duplex. One of the strands or sometimes both represents the functional mature miRNA form that is incorporated in the RNA-induced silencing complex (RISC). This complex is able to target specific mRNAs [26]. MiRBase is one of the databases for miRNA sequences. It permanently increases driven by the deposition of results of small RNA deep sequencing experiments and provides a number of precursor and mature miRNAs that are confirmed based on mapping of small RNA deep sequencing reads [27]. Small RNA sequencing (RNA-seq) data can be analyzed similar to other transcriptome sequencing data based on basic analysis pipelines including quality control, filtering, trimming, and adapter clipping followed by mapping to a reference genome or transcriptome. However, for small RNA-seq data it is necessary to modify the analysis pipeline. The first universal steps of the RNA-seq analysis starting with quality control up to adapter clipping are needed for every FastQ file analysis. For mapping the short miRNA sequences, a specialized mapping strategy is required, e.g., the BWA aligner which works best for well-annotated genomes [28] or the National Center for Biotechnology Information (NCBI) BLASTn-short specifically for short sequences [29]. The universal steps as well as sequence alignment can be performed in Galaxy [30–32].

In human and mouse, a great variation of ncRNA is known compared to other mammalian species. A serious limitation appears when working with incompletely annotated species such as livestock. Since the number of annotated small ncRNAs is relatively low, it is very difficult to comprehensively and accurately annotate small RNA data sets. IsomiRs are miRNA variants that originate from imprecise and alternative cleavage during the pre-miRNA processing and post-transcriptional modifications [33, 34]. This results in different miRNA stability, sub-cellular localization and different target sites [35]. Mature miRNA sequences can have variations at the 3’ or 5’ end or both ends such as additions or deletions. Modifications that do not match the precursor are so-called “non-template” isomiRs and are of particular interest because these isomiRs might indicate active miRNA function, i.e., were used for repression of target mRNAs [36, 37]. Five-prime end isomiRs can have an interesting role because this modification affects the seed region of the miRNA which can lead to a different set of targets [38, 39].

We used small RNA-seq analysis to investigate isomiR expression, expression of blastocyst-specific miRNAs, and effects of a maternal estradiol-17 exposure from fertilization onwards until the day of analysis (Day 10) in single porcine blastocysts.

## Methods

### Animal trial and sampling

The experimental trial has been previously described [6, 40]. In brief, estrous cycle synchronized German Landrace sows (n=4-6/treatment) were inseminated with sperm of the same single Pietrain boar. Starting from insemination until Day 10, sows were fed with different doses of estradiol-17 (E2; 1, 3, 5(10)-ESTRATRIEN-3, 17 -DIOL, Steraloids, Newport, USA), namely with 10 and 1000 µg E2/kg body weight twice daily, respectively, or with 2 ml ethanol carrier only (control group). The E2 concentrations were selected according to reference values for humans referring to oral uptake levels [41] and have been reported earlier [6]. The low dose corresponds to the no observed effect level (NOEL). One hour after ingestion of the last dosing, sows were slaughtered on Day 10 of pregnancy. The uterus was removed and embryos were flushed from the uterus using 10 ml phosphate-buffered saline (PBS) (autoclaved, pH 7.4) per horn. All embryos were transferred into a petri-dish containing PBS, washed twice and then single embryos were snap-frozen in liquid nitrogen and stored at -80 °C. Animals were only included in the RNA-seq analysis if embryos were at the hatched blastocyst stage. Experiments with sows were performed in accordance with the International Guiding Principles for Biomedical Research Involving Animals, as proposed by the Society for the Study of Reproduction, with the European Convention on Animal Experimentation and with the German Animal Welfare Act.

### Extraction of RNA and DNA

Total RNA and DNA from single embryos were extracted as reported recently [12] using the AllPrep RNA/DNA Micro Kit (Qiagen, Hilden, Germany) following the manufacturers protocol for cells with slight modifications. In brief, 700 µl of Buffer RLT Plus supplemented with 1% -mercaptoethanol was added to the frozen embryos. Disruption was achieved by pipetting up and down and by a single brief vortexing. Homogenization was performed using a syringe and needle (20 G). After centrifugation of the lysate using a DNA spin column, the column was stored at 4 °C, while the flow-through was processed following the protocol for “purification of total RNA containing small RNAs from cells”. In order to improve RNA purity, the column was incubated with Buffer RPE at step D3 and D4 before centrifugation for 4 min and 2 min, respectively. RNA elution was repeated using the first eluate to increase the final concentration. The DNA was purified subsequently. Samples were immediately put on ice. RNA and DNA samples were stored at -80 °C and -20 °C, respectively. Purity and quantity was assessed spectrophotometrically using the NanoDrop 1000 (peqLab, Erlangen, Germany). Additionally, RNA quantity of embryos was determined using the Qubit (Invitrogen) with the Qubit™ RNA BR Assay. RNA integrity was measured by means of the Bioanalyzer 2100 (Agilent Technologies, Waldbronn, Germany) with the RNA 6000 Nano Kit (Agilent). The mean RNA Integrity Number of the embryo samples was 9.7 ± 0.3 (± SD).

### Embryo sexing

RNA and DNA, extracted from at least four embryos per sow from four sows per treatment group (control, NOEL, high dose), were used (n = 65). The sex of the embryos was determined by means of quantitative real-time polymerase chain reaction using the DNA with primers specific for the y- chromosomal gene SRY in addition to primers for the autosomal histone gene. Primers were design using NCBI primer-Basic Local Alignment Search Tool (BLAST) [42]. The SuperScript *Ⓡ* III Platinum *Ⓡ* SYBR *Ⓡ* Green One-Step qRT-PCR Kit (Invitrogen) was used on the LightCycler 2.0 (Roche Diagnostics GmbH, Mannheim, Germany). One microliter of DNA was added to 9 µl of the master mix (5 µl of 2X SYBR *Ⓡ* Green Reaction Mix (includes 0.4 mM of each dNTP and 6 mM MgSO4), 2.4 µl of nuclease free water, 1 µl of 20x Bovine Serum Albumin (ultrapure, non-acetylated) (1 mg/ml), 0.2 µl of forward primer [20 µM], 0.2 µl of reverse primer [20 µM], and 0.2 µl of SuperScript *Ⓡ* III RT/Platinum *Ⓡ* *Taq* Mix). PCR was performed with the following thermal cycler program: 50 °C for 10 min, 95 °C for 2 min, followed by 50 cycles for 5 s at 95 °C, 10 s at 60 °C, and 15 s at 72 °C. Melting curve analysis was performed from 55 to 95 °C with 0.1 °C/sec, then samples were cooled to 40 °C. Reactions were run in duplicates for each sample. A positive control DNA was included in each run. Each primer pair was checked for specificity by sequencing the amplification product. The melting point was used for confirmation of the identity of the product for each reaction.

### Small RNA sequencing

NEBNext *Ⓡ* Small RNA libraries were prepared starting from 50–100 ng total RNA from individual embryos (n=6 per group, 6 groups in total) and were sequenced as one pool of 36 barcode-tagged samples on an Illumina HiSeq 4000 (126 bp single-end reads) on one lane. These six groups consists of a control group (ethanol carrier only), low dose (10 µg E2/kg), and a high dose (1000 µg E2/kg) for male and female embryos, respectively. Sequencing was performed at the Functional Genomics Center Zurich and results uploaded as FastQ files to our local Galaxy data storage (Galaxy-Platform version 15.10). NGS experiments have been deposited in EBI ArrayExpress [43] with accession number (E-MTAB-6201).

### Analysis of small RNA datasets derived from porcine endometrium

Two small RNA-seq datasets derived from porcine endometrium were used for comparison of miRNA expression, namely one small RNA dataset for Day 10 of pregnancy generated in a previous study in our group [40], and another dataset for Day 12 of pregnancy downloaded from Gene Expression Omnibus (GSE64863, SRP052027) [13].

### Processing of Fastq files

The data analysis was performed on our locally installed Galaxy system [30]. In the first step of the pipeline, adapter sequences were removed (Illumina Universal Adapter: AGATCGGAAGAGCACACGTCTGAACTCCAGTCAC) with the tool “clip adapter” (Version 1.0.1 FASTX-toolkit [44]). This tool allows to keep clipped sequences and discard non-clipped sequences. Next, Trimmomatic [45], (Version 0.36) was used to remove N bases (any nucleotide, not a gap) at the first position of the 5’ end which affected 15% of all sequences due to a known sequencing issue of the Illumina HiSeq 4000 instrument. All Fastq files were quality checked after each processing step with FastQC (v0.11.2) to control the performance of the processing steps. The data analysis strategy was to generate a count table for all obtained unique sequences. This count table was the basis for the subsequent statistical analysis and to annotate the sequences. For this, a series of standard Galaxy tools were used as well as additional converting tools from the ToolShed [46] (Additional file 1: Figure S1). At first, each FastQ file was collapsed into the sequence and number of appearance (using the tool “Collapse” - FastxTools [44] ranked by the counts (Additional file 2: Figure S2: no. 1). These FASTA files were converted into Galaxy data file type ‘tabular’ (tab-separated text files) with the tool “FASTA-to-Tabular” (Version 1.1.0) (Additional file 2: Figure S2: no. 2). In the following step of the workflow (Additional file 2: Figure S2: no. 3-4) the rank was removed from the table of each identifier generated by the “Collapse” tool followed by two tools (Convert and Cut) to extract the counts and sequence information. With the tool “Convert” dashes were converted into tabs and the columns of interest (counts and sequences) were obtained using “Cut”. In the next step, all separate files corresponding to the individual samples were joined into one table based on the column with the sequences. To join these tabular files, an in-house tool termed “Join datasets by identifier column” was implemented (available in the “ToolShed” Galaxys app store [46] “join_files_by_id”) (Additional file 2: Figure S2: no. 5). The resulting table contained the unique sequences and the number of reads per sample. This count table was filtered to remove sequences with negligible read counts, comprising sequencing errors or sequences with very low evidence for potential expression by using counts per million (CPM) per sample [47]. The mean library size and potential CPM cutoff (Count table statistics, in house tool) was calculated and the cutoff set to 2.64 CPM (corresponding to an average of 20 reads per library) for at least 5 out of 36 libraries.

### Annotation of filtered unique sequences

Filtered sequences were extracted “Tabular to FASTA” and mapped with NCBI BLAST+ [32] blastn-short to align them to all transcripts of *Sus scrofa* including non-coding RNAs and related well-annotated species. The collection of BLAST databases contained sequences from miRBase (precursor and canonical mature miRNAs), transcript sequences from NCBI and Ensembl, including non-coding RNAs, as well as tRNA and piRNA cluster sequences retrieved from NCBI *Sus scrofa* 10.2 GFF3 file [48, 49] (files: mirbase 21: mature/precursor, human, pig, and cattle; Ensembl: predicted precursor sequences, human, pig, cattle, plus other ncRNAs from pig and human; NCBI: all RefSeq transcripts and other non-coding RNAs, for human, pig, and cattle (E-MTAB-6201)). Finally, all BLAST results were filtered and joined by removing all duplicated hits. The annotated sequences were further filtered for potential miRNAs and used for analysis of differential expression. Therefore, we checked if all potential miRNAs also had a significant hit to tRNAs (from pig) or rRNAs (from pig and human) and removed these from the potential miRNAs. Filtering of the alignments was done rather conservative by not allowing any mismatch neither for mapping to porcine nor to the other species (for detailed filtering options see Additional file 3: Table S1).

### Analysis of differential expression

The analysis of differential miRNA and isoform expression was performed with the BioConductor package edgeR [50]. To estimate trended dispersions, first the dataset was normalized on library size (TMM normalized) [50] and the GLM robust (estimateGLMRobustDisp) [51] function was used to dampen the effect of biological outliers. For comparison of the experimental groups, the contrasts (1) male versus female for all estradiol-17 doses and control in separate and (2) low (10) and high (1000) dose versus control separately for male and female embryos were set. An adjusted *p*-value (false discovery rate (FDR)) of 10% and 5% was used as threshold for significance of differentially expressed miRNA sequences. Expression values (CPM) of identified differentially expressed sequences (DES) were further processed with an R script to perform hierarchical cluster (HCL) analysis by samples and DES [52].

### IsomiR analysis

IsomiRs were detected with a pipeline of several R packages from BioConductor [53]. First, all annotated sequences were assigned to their annotated miRNA precursor and aligned with ClustalOmega used by the R package msa [54, 55]. The alignment was used to calculate added and deleted nucleotides and compared to the annotated canonical mature miRNA found in miRBase. In addition, the BLAST result was used to characterize miRNA forms containing non-template nucleotides. Finally, the rank of all isomiRs in their mature miRNA group was calculated according to the individual read counts (expression levels, CPM).

### MiRDeep2 analysis

MiRDeep2 [56] was performed on our locally installed Galaxy system. It allows the user to map small RNA-seq reads against mature miRNA, precursor miRNA and in addition to align all sequences to the corresponding genome. It is also capable of predicting pre-miRNA folding structures of known and novel miRNA genes. Prefiltered Fastq files (quality control and adapter clipping) from our pipeline were used to run first the miRDeep2 mapper (version 2.0.0) followed by the main program to identify novel and known miRNAs. For the comparison, the ARF output file of the miRDeep2-mapper was used which provides all mapped sequences. Additionally, the same miRNA annotation files (see above) were used to map all miRNA sequences.

### Expression database search

The MiRmine - Human miRNA Expression Database was used to search for the expression of human ortholog miRNAs in different tissues [57]. This database provides tissue-specific expression profiles and relative abundance of miRNAs identified in different human miRNA studies.

## Results

### Processing and annotation of small RNA-seq reads

**Fig. 1:**
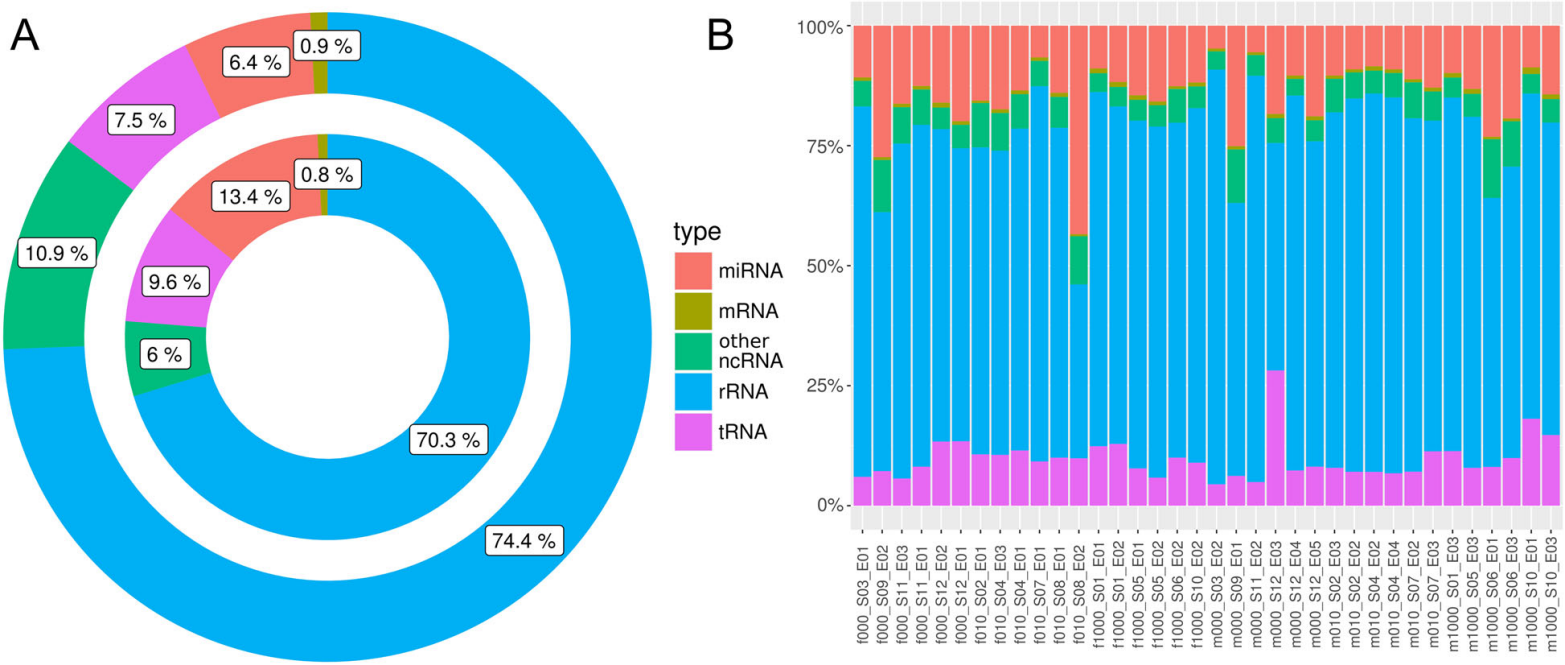
Proportion of mapped reads and read counts: **a** The outer ring shows the percentages of different mapped unique sequences for different types of RNAs. The inner ring represents the percentages of read counts per type of RNA., **b** The proportion of read counts is shown in percent of each type according the different libraries

The sequencing of 36 small RNA-seq libraries for the Day 10 blastocysts resulted in 8 to 32 million raw reads per library. After joining all unique sequences and read counts into one count table for all samples, ∼ 24,000,000 unique sequences were obtained. The count table was reduced to ∼68,000 unique sequences by filtering on >2.64 CPM corresponding to an average of ≥20 reads per sample. Of these sequences, ∼45,000 sequences could be assigned to known RNAs in the annotation files (Additional file 4: Table S2). The majority of the sequences ∼74% (∼70%) (percentage of read counts indicated in parentheses, meaning the number of mapped sequences per unique sequence) mapped to rRNAs, ∼6% (∼13%) to miRNAs, and ∼8% (∼10%) to tRNAs, less than 1% (<1%) of the sequences were assigned to piRNA clusters and ∼1% (<1%) to mRNAs (Fig. 1a). The remaining 11% (∼6%) of sequences were mapped to other groups of ncRNAs. With respect to read counts per RNA type we found large differences between samples (Fig. 1b, Additional file 5: Figure S3). For example, libraries varied from ∼ 6% to ∼42% in miRNA sequence content with respect to read counts.

### Analysis of miRNA using a galaxy pipeline

The predominant length of all annotated miRNAs was ∼23 nt ranging from 16 nt for small isomiRs and 62 nt long sequences mapping to precursor miRNAs. In total, 2949 unique sequences were assigned to miRNAs of miRBase21 using all mature and precursor miRNAs from *Sus scrofa*, *Bos taurus* and *Homo sapiens* (Additional file 6: Table S2). Of these, 1842 (62%) sequences mapped to known miRNAs of *Sus scrofa*. In addition, we detected 1107 (38%) sequences for novel porcine miRNAs, 443 (15%) mapping to *Bos taurus* and 623 (21%) *Homo sapiens*, and 41 (∼1%) to predicted Ensembl porcine novel miRNAs (Additional file 7: Figure S4). Overall, 71 of these novel miRNAs had also been assigned to a porcine pre-miRNA, which indicates that they might not be novel miRNA genes, but novel mature miRNAs. One-hundred-and-nine sequences were filtered out because although annotated as porcine miRNAs in miRBase, they were highly similar to human rRNAs. Another 17 sequences were removed due to their positive hit to porcine tRNA sequences (Additional file 3: Table S2). Altogether, the sequences reliably assigned to miRNAs represented 257 mature miRNAs. Of these miRNAs 162 were detected as known porcine miRNAs (miRBase21) and 95 were novel miRNAs for the pig which were assigned to known miRNAs from human and cattle (Additional file 6: Table S3).

### Comparison of galaxy pipeline to miRDeep2

**Fig. 2:**
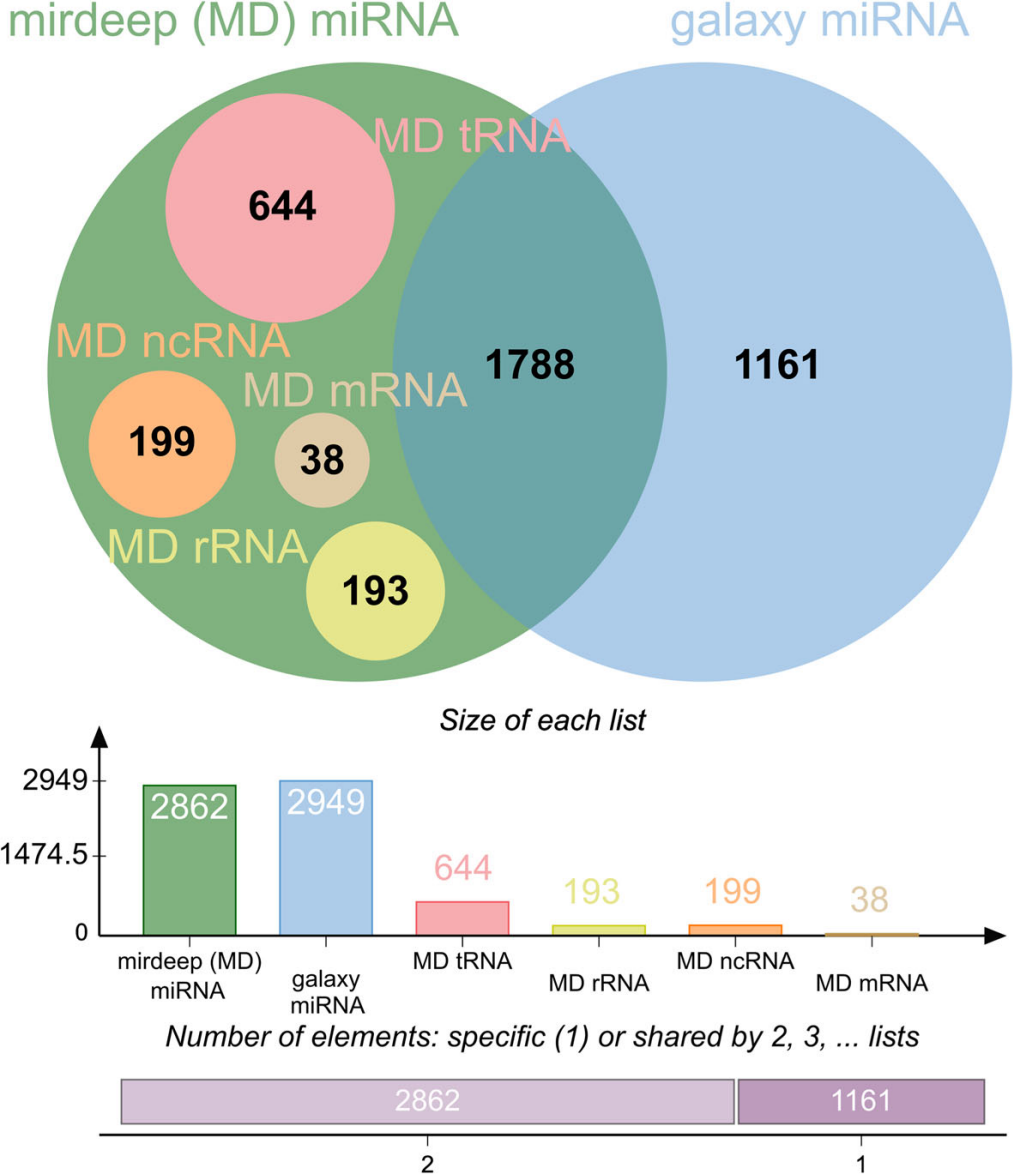
Pipeline comparison: The Venn Diagram shows the overlap between our galaxy pipeline and the MirDeep2 pipeline on the level of detected unique sequences [86]

The focus of the comparison of our pipeline to miRDeep2 was on mapping all reads to mature and precursor miRNAs and compare the differences in isomiR content and expression levels. Therefore a pre-result file including all mapped miRNA isoforms was used within the miRDeep2 pipeline (Result file of miRDeep2.pl “Text output of MiRDeep2”). MiRDeep2 detected 2862 potential miRNA sequences after filtering on low expressed miRNAs (5 out of 36 libraries > 20 reads each which is equal to the overall expression of equal or greater 180 reads in total). Of those, 1788 were overlapping with our pipeline. Exactly 1074 sequences were assigned to miRNAs by miRDeep2 and identified as false positives, 644 mapped to tRNAs, 193 to rRNAs, and 38 to mRNAs. In addition, miRDeep found 199 miRNAs, which could not be mapped to known miRNAs but to other ncRNAs. Of the miRNA sequences, 1161 were uniquely annotated by our pipeline (Fig. 2). With respect to expression levels, no significant differences in reads counts were detected in comparison to miRDeep2.

### Analysis of differential miRNA expression in response to maternal estradiol-17 treatment and between female and male embryos

**Fig. 3:**
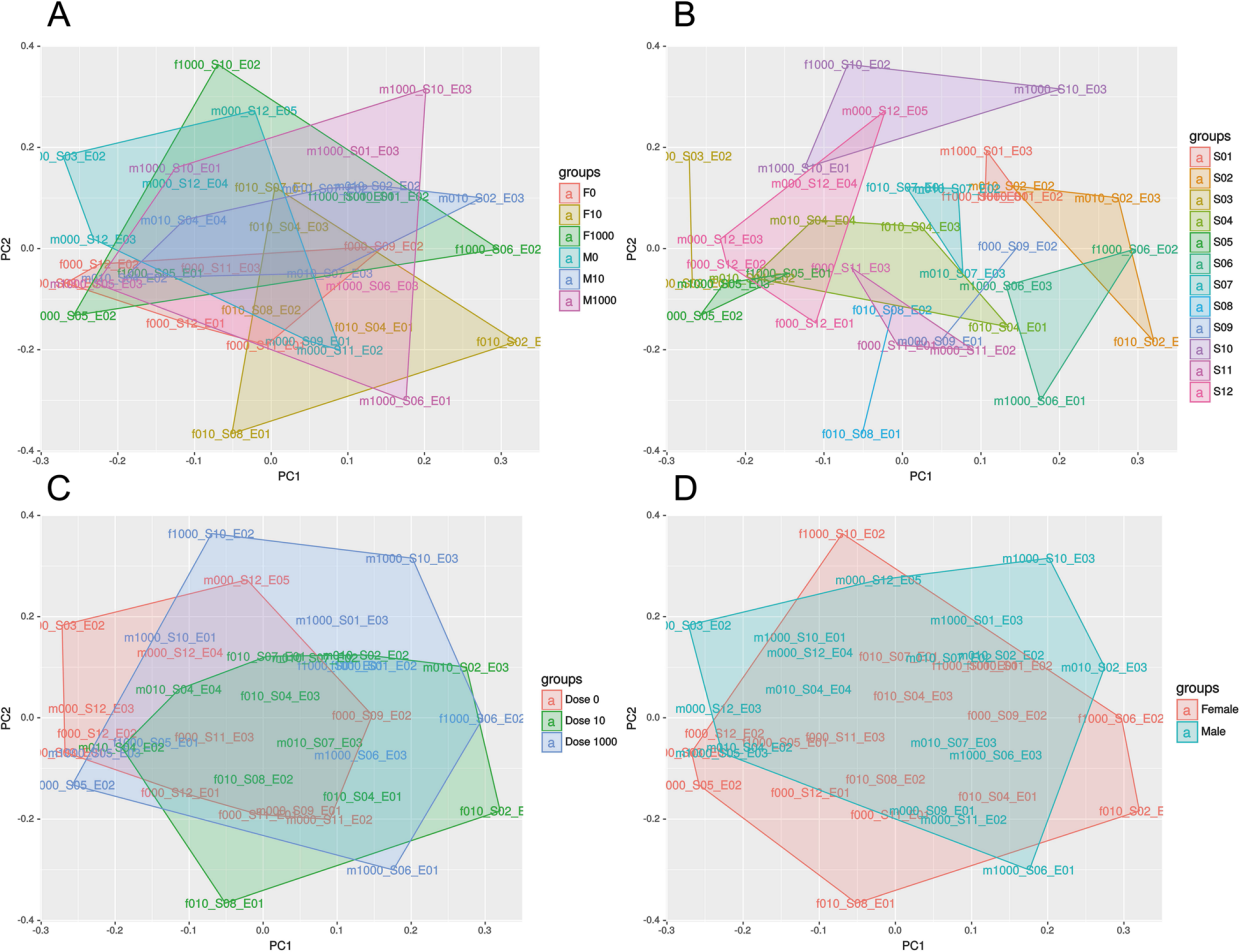
Multiple scaling plot of the top 200 on isomiR level: showing **a** the different treatment groups of male and female embryos with dose 0 (control), 10 (low) and 1000 (high), respectively (ng/µg E2 per kg body weight per day), **b** the grouping of the mother sows, **c** the grouping of the different doses and **d** the grouping of female and male sex

**Fig. 4:**
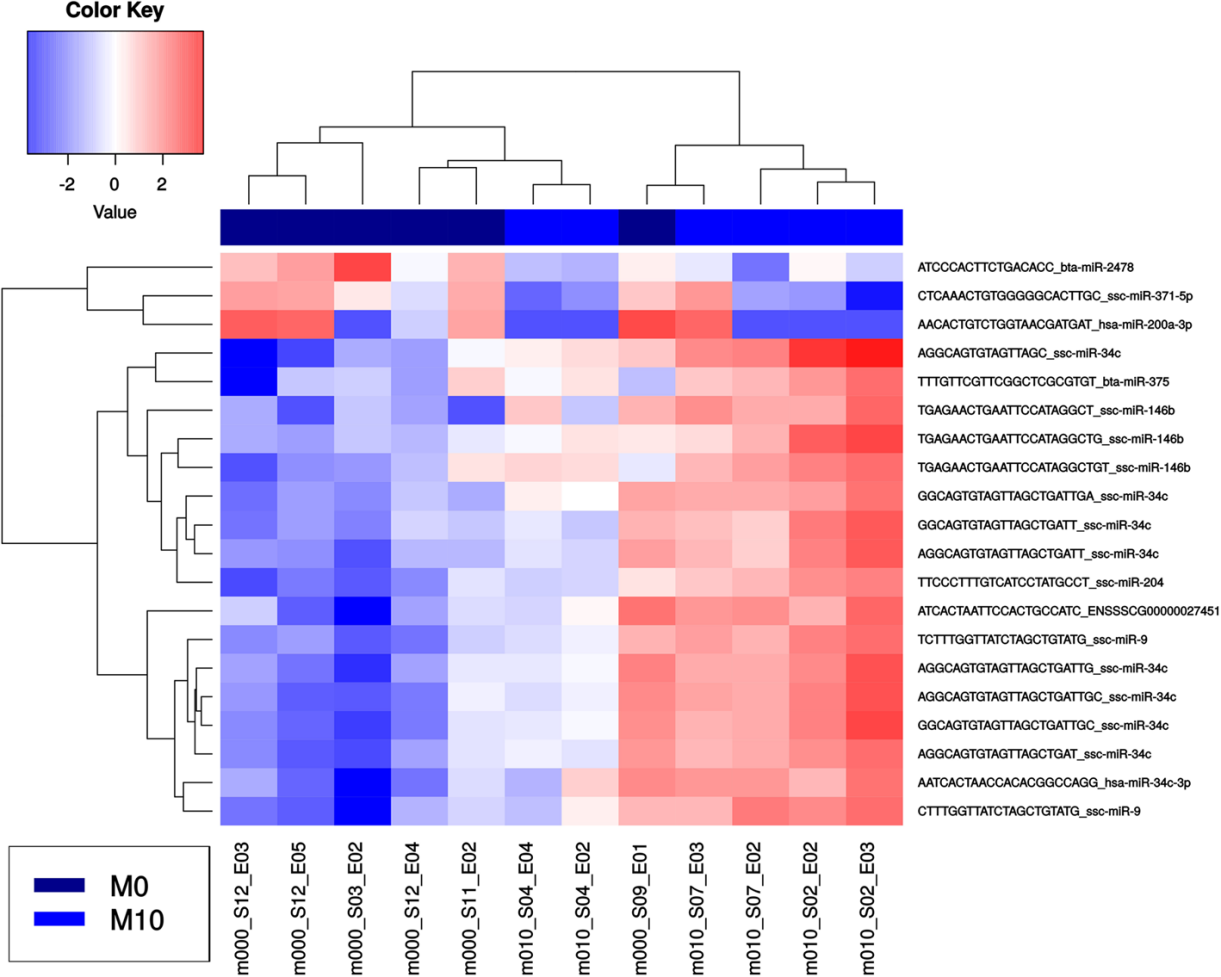
Heatmap of male low dose versus male control treatment (FDR 0.05): The x-axis shows the different samples of the two compared groups M0 and M10 (M0: male embryo control group; M10: male embryos low dose group). The y-axis shows the potential differentially expressed sequences (isomiRs) obtained in the respective comparison. Mean-centered log2 CPM values are shown

For the statistical analysis of miRNA expression, a filtered count table was used containing 2892 annotated miRNA sequences. None of the generated multiple scaling plot (MDS) plots showed a grouping of embryo samples neither by treatment nor by sex (Fig. 3a, c, d, Additional file 8: Figure S5). A grouping of embryos collected from the same sow was observed in the MDS plots (Fig. 3b) and in the pairwise distance heatmap (Additional file 9: Figure S6). The comparison of male versus female embryos did not reveal DES (FDR 5%). The comparison of low dose estradiol-17 feeding versus control resulted in 20 DES for male embryos (FDR 5%), which represented 10 different mature miRNAs. The miRNA with the highest number of DES was ssc-miR-34c. All other comparisons did not detect DES (Additional file 10: Table S4). A HCL analysis was performed for these 20 DES to characterize the homogeneity of the expression within the treatment groups (Fig. 4). The cluster analysis did not reveal a clear grouping according to treatments and control. In addition, the read counts of isomiRs were summarized for each miRNA and also female and male embryo samples were joined for each treatment and the control group to increase the number of replicates. Furthermore, the embryos nested in the sow and further technical batch effects were included in the statistical model. None of the various analyses revealed more reliable significant miRNA expression differences.

### Analysis of tissue-specific miRNA expression

**Fig. 5:**
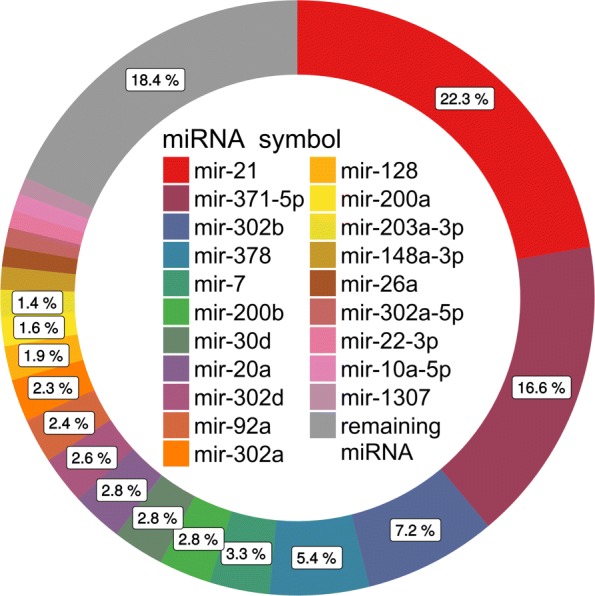
Embryonic miRNA: Top 20 expressed miRNAs: The percentage of read counts assigned to the top 20 expressed miRNAs in addition to the sum of all remaining miRNAs

**Fig. 6:**
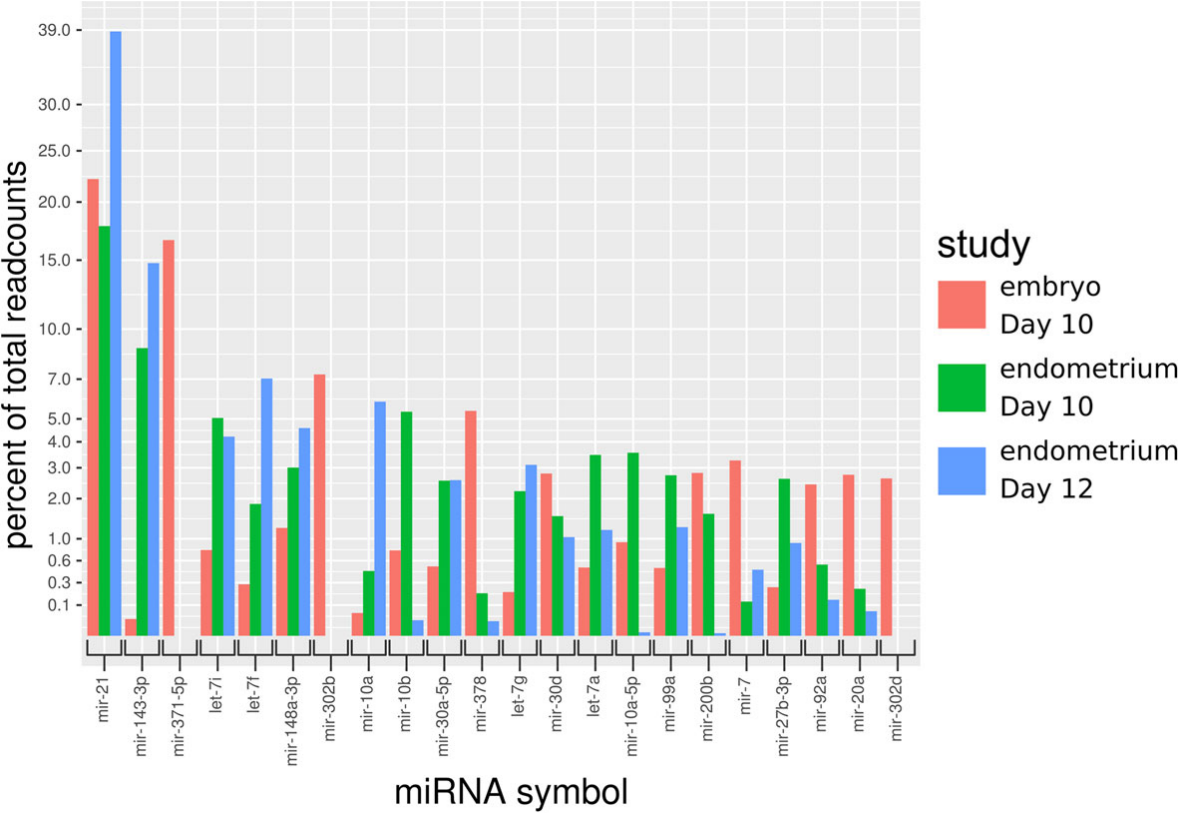
Tissue comparison: Top 10 expressed miRNAs in embryo Day 10, endometrium Day 10 and 12

To identify embryo-specific miRNAs potentially involved in regulation of development, the embryo miRNA expression results were compared to two datasets from porcine endometrium and to a miRNA expression database (MiRmine). The data sets were analyzed with the same pipeline and follow-up scripts. The expression levels of the top 20 most frequently expressed miRNAs in the embryo were compared. All three datasets had mir-21 (miR-21-5p) as the most highly expressed miRNA with 39% of all reads in Day 12 endometrium, 18% in Day 10 endometrium and 22% in Day 10 embryos (Additional file 11: Table S5, Fig. 5). In addition to commonly expressed miRNAs, potential tissue-specific miRNAs were identified. For this, we compared the top 10 expressed miRNAs of each study, which resulted in 22 different miRNAs. For endometrium Day 10 or Day 12, mir-143-3p (∼12% of all reads), let-7i, let-7f, and let-7g (each ∼4% of all reads), mir-10b and mir-10d (each ∼5%) were classified as potential embryo-underrepresented miRNAs. Five miRNAs were identified as potential embryo-enriched miRNAs (mir-371-5p 16%, mir-302b 7%, mir-378 ∼5%, mir-7 ∼3%, and mir-302d ∼3%) (Fig. 6). These results were further validated by a search in the miRmine miRNA expression database, which contains expression data for human miRNA for 15 different tissues collected from a variety of deep sequencing studies. The results showed that miR-371-5p is also highly expressed in placenta and umbilical plasma, and at considerable levels in testis, semen/sperm, and salivary exosomes. Furthermore, high expression was found in tumor tissues. MiR-302b-5p and miR-302d-5p were not found as expressed in any dataset contained in miRmine. MiR-378-5p is highly expressed in umbilical plasma, blood macrophages, and other plasma and serum samples. Lower expression was also found in testis, placenta, and a variety of tumors. MiR-7-5p expression in the miRmine data sets was very high in pancreas beta cells and high in various plasma samples, testis, sperm, placenta, serum, salivary exosomes, CD4+ T cells and CD19+ B cells from blood, and different tumor tissues. The most dominant potential endometrium-specific miRNAs (mir-148a-3p, let-7i, mir-30a-5p, let-7g, mir-143-3p) were all equally high expressed in all 15 tissue types (Additional file 12: Table S6). MiR-21-5p was highly expressed in all tissues, which confirmed our findings from the comparison of embryo and endometrium.

### IsomiR expression analysis

**Fig. 7:**
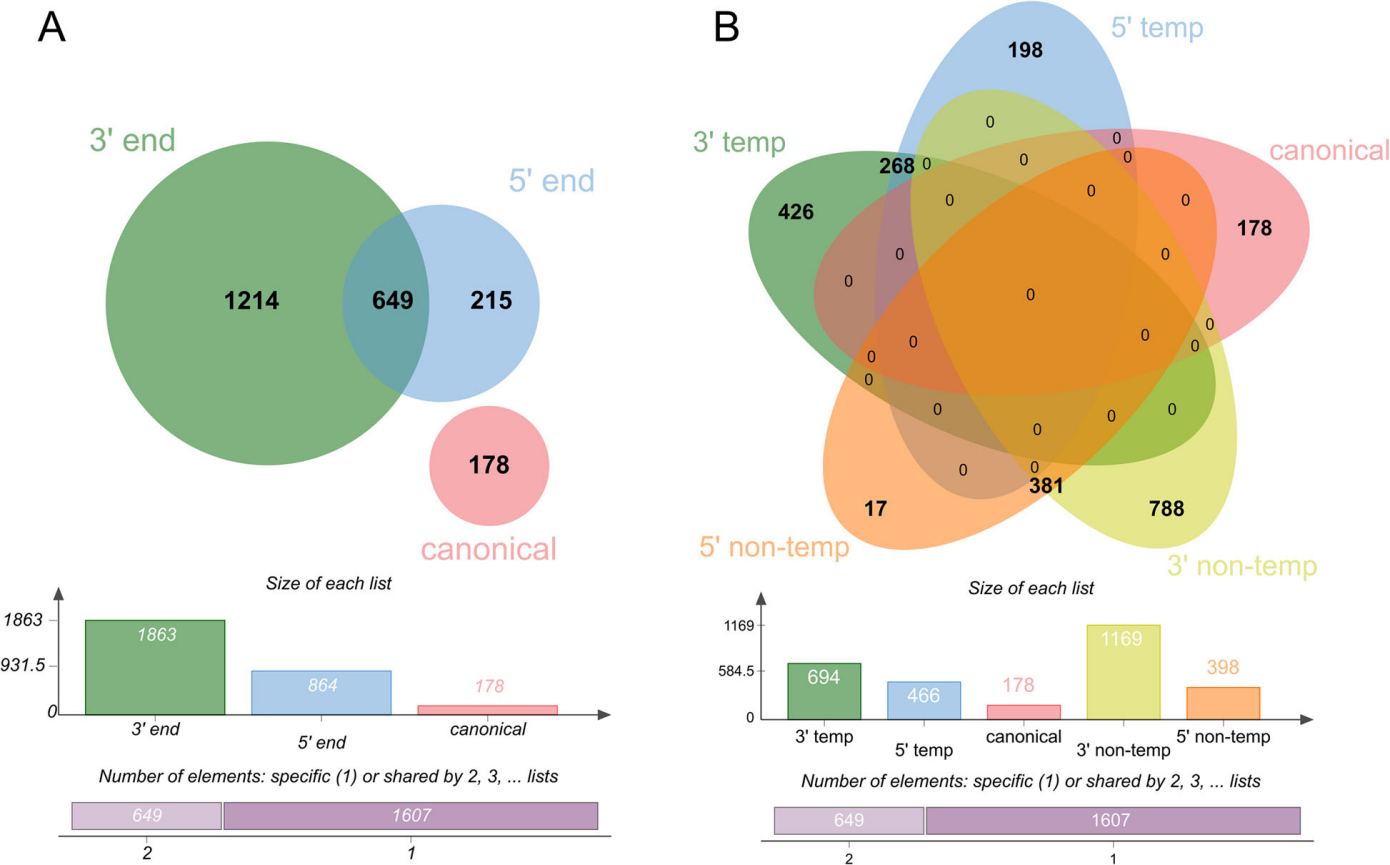
Detected miRNA isoforms (isomiR): The Venn Diagram on the left (**a**) represents the number of different detected isomiR on sequence level organized by the modification types. The Venn Diagram on the right (**b**) represents the number of different detected isomiRs on sequence level split into templated and non-templated forms

**Fig. 8:**
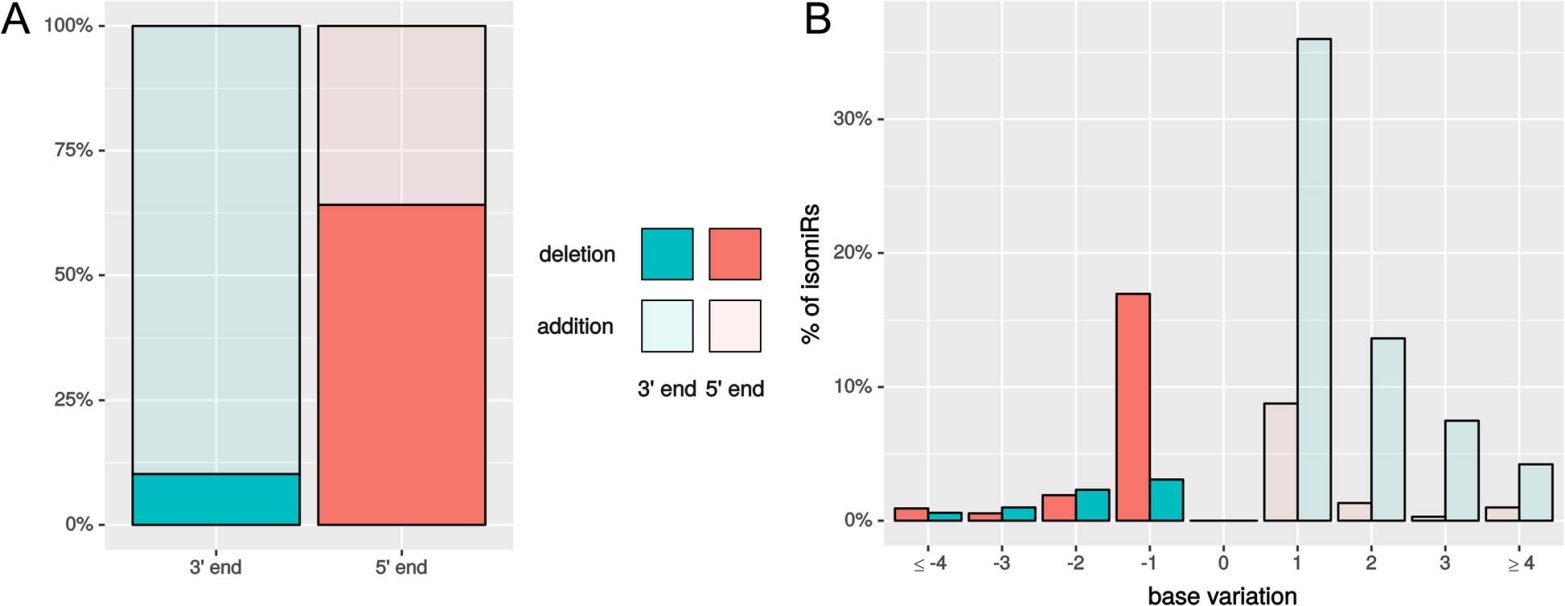
MiRNA modification differences: The barplot on the left (**a**) shows the percentage of isomiRs assigned to additions and deletions separated by 3’ and 5’ end. The right (**b**) barplot represents the base variations of added and deleted bases for all detected miRNAs at 3’ and 5’ end

**Fig. 9:**
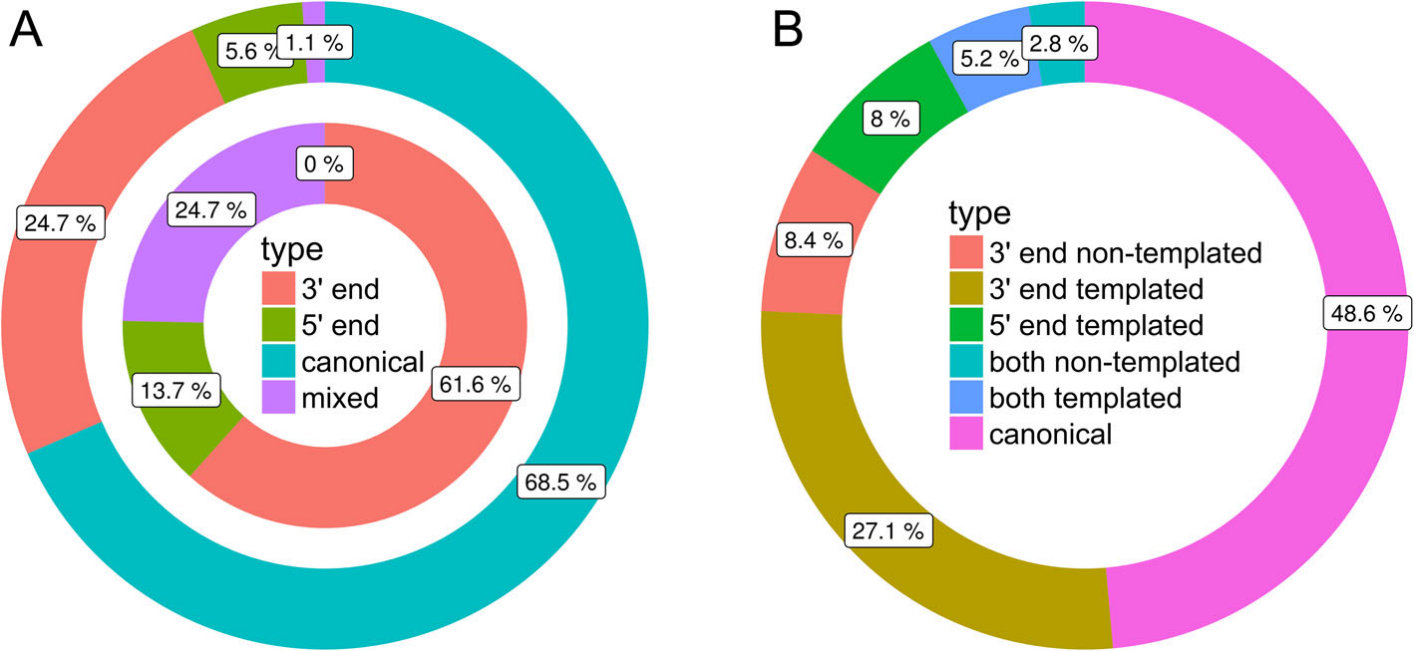
Top expressed isomiRs: The donut plot on the left (**a**) represents the top (rank 1) expressed isomiRs per miRNA group. The outer cycle shows miRNA groups containing the canonical form (mature form of the database) and the inner ring are miRNA groups without the canonical form present. The plot on the right (**b**) shows the top expressed isomiRs on sequence level organized by templated versus non-templated

The top 30 highly expressed miRNAs represented ∼89% of all read counts and the top 4 miRNAs ∼51%. Of those, miR-21 was the most prominent, accounting for ∼22% of all miRNA read counts (Additional file 13: Table S7, Fig. 5). On average, 5 isomiRs per mature miRNA were detected (from 1 to 138). The greatest variety of different isomiRs were expressed by ssc-miR-371-5p (138) and ssc-miR-21 (67) (Additional file 14: Table S8). Modification only at the 3’ end occurred with 54% (1214), while 29% (649) had a modification at both sides and 10% (215) only at the 5’ end. The canonical form was found for 178 miRNAs (Fig. 7). The variations from the canonical form found in miRBase were most frequently added bases rather than deletions (Fig. 8a). At the 5’ end, most frequently one deletion or one addition were found (Fig. 8b). At the 3’ end, one or two added nucleotides per isomiR occurred most frequently (Additional file 14: Table S8, Fig. 8a). Regarding the expression levels per isomiR, mostly the canonical form was most abundant (∼68%), closely followed by isomiRs with 3’ end modifications (25%) (Fig. 9a). For miRNAs where the canonical form was not present (89 miRNAs), the highest expression level was detected for 3’ end modifications (∼ 61%), followed by modifications at both sides (25%) and modifications at the 5’ end (∼14%). Furthermore, the expression levels of isomiRs were analyzed. For the expression analysis on isomiR level, low (on average less than 20 reads per sample) and moderate up to high expressed isomiRs were defined (more than 20 reads per sample). In total, 1003 isomiRs were identified with low expression (Additional file 15: Table S9) and 1946 sequences as highly expressed. The isomiRs containing non-template nucleotides sequences could be detected at 5’ end (398), at 3’ end (1169) and or sequences that had modifications on both sides (381) (Fig. 7b). The analysis of expression levels of all non-templated miRNAs revealed that 28 sequences with ∼10% of the 3’ end non-template isomiRs and ∼ 3% of the 5’ non-template isomiRs showed the highest expression of the corresponding miRNA (Additional file 14: Table S8, Fig. 9b). The top 5 most frequently expressed miRNAs were hsa-miR-200a-3p, bta-miR-21-5p, bta-miR-378d, bta-miR-1246, and ssc-miR-210 ranging from ∼6000 till ∼62,000 read counts equally distributed through all samples.

## Discussion

In this study, we used core Galaxy tools complemented with customized scripts to make the analysis of porcine small RNA as straightforward as possible. We specifically included sequences of well-annotated species from various sequence databases to improve the annotation of the sequences found in the small RNA-seq results. This strategy allowed the identification of sequence fragments derived from rRNAs and tRNAs as well as other ncRNAs or mRNAse. Furthermore, a strategy to analyze variant isoforms of miRNAs (isomiRs) was developed [13, 58], increasing the percentage of annotated sequences and thereby adding additional information to the subsequent statistical data analysis of each expressed small RNA.

Earlier studies aimed at standing the role of differential miRNA expression patterns in embryonic and fetal preimplantation development and in embryo/feto-maternal interactions in pigs [13, 15, 59–62] (Flöter et al, under review). In contrast, the present study focused on the hatched blastocyst prior to conceptus elongation and specifically explored effects of a maternal estradiol-17 exposition on embryonic miRNA patterns. To our knowledge, this is the first study investigating sex-specific miRNA profiles including isomiRs of single preimplantation embryos.

### Development of a small RNA-seq analysis pipeline for incompletely annotated species

The pig served as large animal model for the effect of endocrine disrupting chemicals in humans. Compared to rodents, it better resembles the women regarding the endogenous placental estrogen production during fetal development [6]. However, working with incompletely annotated species such as the pig makes the analysis of small RNA-seq data sets much more difficult compared to human or mouse. In pig, the miRNA annotation is rather incomplete with respect to the relatively low number of annotated miRNAs (342) that can be found in miRBase (version 21). Therefore, a corresponding analysis pipeline requires an approach that is focused on annotation of new miRNAs. The approach used in the present study is based on sequence comparison (BLAST) of the sequences obtained from small RNA-seq to various sequences derived from the pig, next to different types of transcripts from cattle and human. Since the generation of small RNA-seq libraries does not specifically select miRNA sequences [63], further potential source sequences have to be included such as rRNAs, tRNAs, and other ncRNAs in addition to the available precursor (pre-) and mature miRNAs. Particularly, fragments of tRNAs, rRNAs or other small ncRNAs have been falsely identified as miRNAs based on their respective size [64, 65]. The comparison of the results of the new analysis pipeline to a standard miRNA analysis pipeline (miRDeep2) revealed differences and some limitations, mainly on the side of miRDeep2. In particular, the mapping to other ncRNAs including tRNAs in the new pipeline turned out to be a great improvement to prevent misannotation of miRNAs. Both tools detected similar numbers of miRNA sequences, but only 62% of the sequences assigned to miRNAs were overlapping. The analysis of the non-overlapping sequences revealed that 1074 potential miRNA sequences from the miRDeep2 tool were derived from other ncRNAs such as rRNAs, tRNAs or other ncRNA classes. Other studies also noticed the problem of wrongly annotated miRNAs that were actually derived from tRNAs [64, 65]. Venkatesh et al. stated that such sequences would represent two new classes of regulatory non-coding small RNAs that are derived from the cleavage of pre-existing tRNAs, namely tRFs and tiRNAs. Schopman et al. (2010) concluded that such small RNA fragments described as miRNAs in miRBase are likely derived from tRNA processing. In our study, the respective sequences were present in a size range of miRNAs up to about 100 nt and were all covering the same tRNA sequence. Schopman et al. (2010) also concluded that the rapid release of data from small RNA-seq projects would lead to a misannotation of miRNAs in databases such as miRBase. Especially sequences derived from fragments of rRNAs and tRNAs are often highly abundant and present in varying percentages in small RNA libraries. This might introduce a severe bias in estimating miRNA expression levels and thereby affecting the detection of differentially expressed miRNAs.

### Effects of a maternal estradiol-17 treatment and of the sex of the embryo on miRNA expression in blastocysts

We recently demonstrated that the maternal low-dose estradiol-17 treatment led to a perturbed endometrial mRNA expression profile of sows as well as their developing blastocysts [12] (Flöter et al, under review). This is of particular note, as low estradiol-17 doses, considered to exert no effect, were applied [6]. Because we found a shift in body composition [6] and bone density [11] in male and female offspring, respectively, we considered a sex-specific intergenerational epigenetic epigenetic reprogramming imposed as early as the preimplantation phase. Recently, it has been shown that the biogenesis of uterine miRNAs is steroidal regulation [66]. Therefore, we hypothesized that the maternal oral E2 exposure could lead to an inference with normal miRNA expression in the early embryo. This could be attributed to a direct estradiol-17 impact on the embryo on the one hand or through perturbations of the endometrial secretions due to the estrogen treatment leading to changes in the uterine milieu (Flöter et al, under review), that could in turn have effects on the developing embryo.

Similar to the analysis of miRNA expression in the endometrium samples (corresponding to the samples of the present study), where miRNA expression was not affected by E2 treatment, only small effects were found on male Day 10 embryos. Since only a very low number of miRNA sequences showed significant differences in male embryos of the low dose group compared to controls and the biological replicates did not group very well according to treatment or sex in the cluster and principal component analysis, it is difficult to say if these observations are true expression differences in response to the E2 feeding. Furthermore, despite some expression differences were found in male embryos, differential miRNA expression between female and male embryos was not detected. The observed clustering of embryos collected from the same sow is most likely attributed to a maternal effect on sibling embryos. In addition, a similar developmental stage for siblings can be assumed, since although the time point of fertilization varies between sows, it is almost the same for siblings within the same sow [67]. However, considering the sow as batch effect in the statistical analysis did not change the results. It is possible that not every sow and/or embryo was affected by the treatment in the same way. However, we did not find a difference in variation among the control and the treatment groups. Overall, from the obtained results we can say that there is only a slight effect of the maternal estradiol-17 feeding on miRNA expression only in male embryos.

With respect to the experimental model, the time point of the analysis may limit a generalization of the finding. The sample collection was performed one hour after the last estrogen dose, which might have been too late to observe a very rapid miRNA response. On the contrary, it is possible that the former ten days of treatment might have led to an adaption of miRNA transcription. Further studies are needed to verify the present findings. This adaption might account for the difference to an earlier study in which the singular exposure to estradiol-17 at days 9 or 10 of pregnancy resulted in pregnancy failure [16]. The pregnancy rate in our study, giving a continuous treatment twice daily from fertilization until Day 10, was not affected, not even in the high dose group [40]. The treatment mode, namely i.m. injection versus oral application in our study, most likely lead to a different steroid metabolization possibly exerting the particular lack of an effect in our case. It thus remains to be further unraveled which circumstances estrogen treatment does not affect the miRNA expression of a target tissue.

Interestingly, a sex-specific differential miRNA expression between embryos was likewise excluded. The HCL analysis showed a partial clustering of embryos collected from the same sow, which is most likely attributed to a maternal effect on siblings embryos. In addition, a similar developmental stage for siblings can be assumed, since although the time point of fertilization varies between sows, it is almost the same for siblings within the same sow [67].

### Identification of potential embryo-specific miRNAs

The comparison of the embryo miRNA profiles to small RNA-seq studies in porcine endometrium collected on Day 12 [13] and Day 10 of pregnancy [40] identified not only miRNAs with similar expression levels for some of the most abundant miRNAs, but particularly also tissue-specific miRNAs. Both endometrium and embryo samples are mixed tissues containing many different cell types such as epithelial cells, fibroblasts, endothelial cells, and various immune cells in the endometrium, and trophectoderm and embryoblast/hypoblast cells in the Day 10 blastocyst [68]. MiR-21 was highly expressed in both tissues and the most abundant miRNA in the embryo as well in Day 10 endometrium. A high expression of miR-21 was also reported in other studies in porcine embryos [69], oocytes [15], embryos of other species [14], and in a variety of somatic tissues as well as cancer cells [70, 71].

Four miRNAs (miR-371-5p, miR-302a, miR-302b, and miR-302d) were expressed only in blastocysts and not in endometrium. MiR-371-5p is located within a pregnancy-related miRNA cluster [72], and its increased expression has been found in the first compared to the third trimester human placenta [73]. A database (miRmine) search revealed its high expression in 9 (bladder, brain, breast, placenta, plasma, saliva, semen, sperm, and testis) out of 15 tissues including placenta, which indicates a tissue-specific expression. Mir-302b was not expressed in any tissue contained in the database, which supports our finding from the comparison to the endometrium that miR-302b could be embryo-specific. MiR-371 and miR-302a,b,d have been found specifically expressed in human embryonic stem cells [74]. In the mouse, for the homolog miRNA gene cluster to the human mir-371-373 cluster (mir-290-295) and the mir-302 cluster, specific expression in early embryos and embryonic germ cells was found, and an important role in pluripotency and embryonic development has been suggested [75–77]. An upregulation of miR-302a, miR-302-b, and miR-371-5p was found in porcine induced pluripotent stem cells in comparison to porcine embryonic fibroblasts [35]. In the same study, the mir-302 gene cluster has been found to improve reprogramming efficiency. On average we detected 5 isomiRs per miRNA. Interestingly, for mir-302b, mir-302d, and mir-302a, which are not yet contained in the miRBase for the pig (miRBase 21), more different isoforms than the average were found (mir-302b (23), mir-302d (33), and mir-302a (42). All potential endometrium-specific miRNAs (mir-148a-3p, let-7i, mir-30a-5p, let-7g, mir-143-3p) were highly expressed in all 15 tissue types included in the miRmine database. This indicates that these miRNAs are not truly endometrium-specific. As the endometrium is a complex tissue comprising epithelial cells, fibroblasts, endothelial cells, and various immune cells, it remains to be determined in which of the endometrial cell types these miRNAs are actually expressed.

### Analysis of isomiR expression in blastocysts

The analysis of isomiRs was performed according to a previous study investigating retina samples [78] to identify templated and non-templated variations at the 5’ and 3’ end. Therein, more variations in form of deletions in comparison to the canonical form rather than added bases have been found. Mostly, the isoforms were one base shorter than the canonical which occurred more often at the 5’ end. In contrast, the embryos displayed more added bases and in general more variations at the 3’ end. These could play a role in the effectiveness of miRNA targeting. Specifically, 3’ non-templated adenylation of the mature miRNA has been shown to interfere with incorporation into the RISC [79], whereas monouridylation of the pre-miRNA is required for processing by Dicer for group II let-7 miRNAs [80] and oligouridylation, in contrast, blocks uptake into Dicer [81, 82]. Adenylation at 3’ non-templated miRNAs tend to be higher with ∼35% compared to the other nucleotides C, G, and U with ∼20% whereas modifications of uridine could not be detected.

In addition, the distribution of expression level of different isomiR types (canonical, templated, 3’ and 5’ modifications) was analyzed for the highest expressed miRNAs. In accordance with Karali et al. (2016), the isomiR showing the highest expression was mostly the canonical or templated form. Furthermore, in studies of human embryonic stem cells [38] and human hepatocellular carcinoma [83] different isomiRs resulting from deletions and additions at the 5’ and 3 ’ side were identified and characterized. Consistently, the authors found more modifications at the 3’ end compared to the 5’ end and concluded that this was due to the greater constraint of the 5’ side. This finding is also supported by the possibility of shifting the seed region [84, 85], which can change the target mRNA spectrum.

In our analysis, we found a high number of non-templated isomiRs of rather low expression level. Non-templated isomiRs at the 3’ and 5’ end were only highly abundant in ∼11% of all cases (rank 1). Due to the possible target change they are often not dominantly expressed [83]. As a limitation of our data set, most of the deletions of one nucleotide at the 5’ end were caused by the removal of the first position of the obtained sequence reads during quality trimming, since a “N” occurred at the first position in 15% of all sequences. This issue was derived from running the samples on the Illumina HiSeq 4000 instrument. However, five-prime non-templated events may turn out to be of special interest in case they can be shown to have a modified seed sequence and thereby could have a different range of target transcripts.

## Conclusion

In conclusion, despite the lack of both sex-specificity and the significancy of effects of a maternal estrogen treatment on miRNA expression in blastocysts, the deep sequencing of small RNAs in porcine blastocysts revealed a high variety of expressed miRNAs and their isomiRs. A catalog of isoforms of known porcine miRNAs and many novel porcine miRNAs was generated indicating the high complexity of miRNA expression and regulation in pre-implantation embryo. Their role in embryo development specifically related to the observed robustness towards the maternal estrogenic treatment requires further exploration.

## Additional files

Additional file 1 **Figure S1.** Complete analysis pipeline in Galaxy: 1) Quality control and trimming plus adapter clipping. 2) MiRNA pipeline to convert sequences into a count table. 3) Filtering count table and sequence identification. (PNG 828 kb)

Additional file 2 **Figure S2.** Schema of the Galaxy pipeline: 1) to collapse FastQ file into the sequence and number of appearance ranked by the counts. 2) converts FASTA files into the Galaxy data file type “tabular” (tab-separated text files). 3) converts dashes in to tabs and 4) extracts selected columns of a given tabular file. 5) joins files by a selected identifier column. (PNG 4714 kb)

Additional file 3 **Table S1.** Filtering details for potential miRNAs. (XLSX 73 kb)

Additional file 4 **Table S2.** Blast result with all hits to database files found (E-MTAB-6201). (XLSX 2127 kb)

Additional file 5 **Figure S3.** Library sizes of mapped reads per RNA type. (PNG 617 kb)

Additional file 6 **Table S3.** Blast result with only hits related to miRNA features. (XLSX 246 kb)

Additional file 7 **Figure S4.** Known and novel miRNAs and species annotation - unique sequences and read counts. In the outer ring the mapped unique sequences are shown related to mapped species. The inner cycle represents the corresponding read counts per species. (PNG 468 kb)

Additional file 8 **Figure S5.** MDS plots of the top 300 and 2000 isomiRs. The different colors represent the six different treatment groups (three different E2 doses with respective male and female embryos). (PNG 533 kb)

Additional file 9 **Figure S6.** Distance heatmap of all samples. (PNG 1065 kb)

Additional file 10 **Table S4.** EdgeR result of all comparisons on isomiR level. (XLSX 32902 kb)

Additional file 11 **Table S5.** Embryo Day 10 miRNAs expression versus endometrium Day 10 and Day 12. (XLSX 70 kb)

Additional file 12 **Table S6.** MiRmine result of potential embryo- and endometrium-specific miRNAs. (XLSX 67 kb)

Additional file 13 **Table S7.** Expression level embryo Day 10 in percentage. (XLSX 7 kb)

Additional file 14 **Table S8.** IsomiR analysis. (XLSX 202 kb)

Additional file 15 **Table S9.** IsomiR count table. (XLSX 594 kb)

## Abbreviations

BLAST: Basic Local Alignment Search Tool
CPM: counts per million
DES: differentially expressed sequences
E2: estradiol-17
EDC: Endocrine disrupting chemical
FDR: false discovery rate
HCL: hierarchical cluster
pre-miRNA: precursor hairpin
piRNA: PIWI-interacting RNA
pri-miRNA: primary precursor
MDS: multiple scaling plot
miRNA: microRNA
mRNA: messenger RNA
NCBI: National Center for Biotechnology Information
ncRNA: non-coding RNA
NOEL: no observed effect level
PBS: phosphate-buffered saline
RISC: RNA-induced silencing complex
RPM: reads per million
RNA-seq: RNA sequencing
rRNA: ribosomal RNA
tRNA: transfer RNA
