# Supplementary figures and images for: Small RNA-seq analysis of single porcine blastocysts revealed that maternal estradiol-17beta exposure does not affect miRNA isoform (isomiR) expression
Source: BMC Genomics. 2018 Aug 6;19:590. doi: 10.1186/s12864-018-4954-9 (PMC6090871; doi:10.1186/s12864-018-4954-9)

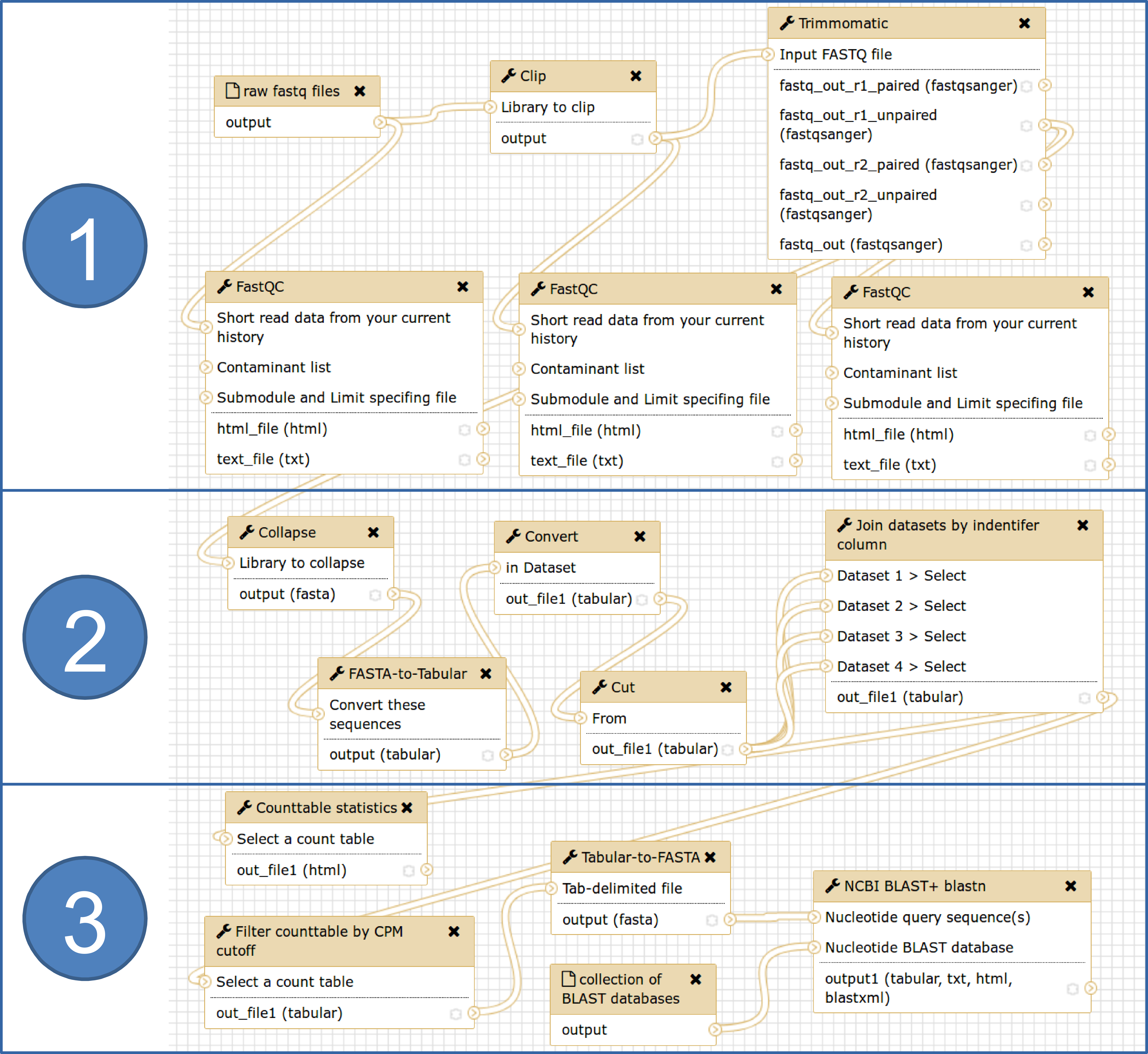

Supplement: Supplementary file 1 — Figure S1. Complete analysis pipeline in Galaxy: 1) Quality control and trimming plus adapter clipping. 2) MiRNA pipeline to convert sequences into a count table. 3) Filtering count table and sequence identification. (PNG 828 kb) [file 12864_2018_4954_MOESM1_ESM.png]

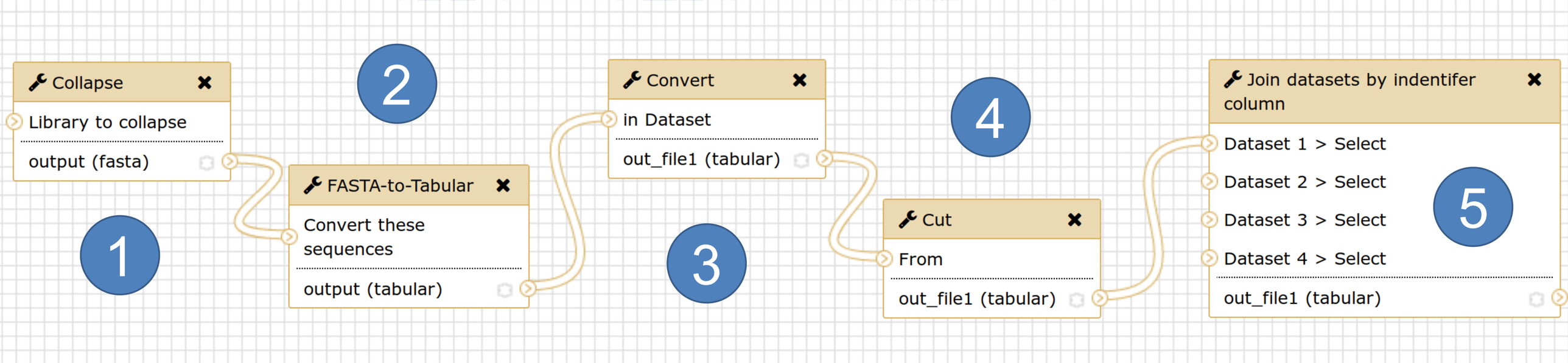

Supplement: Supplementary file 2 — Figure S2. Schema of the Galaxy pipeline: 1) to collapse FastQ file into the sequence and number of appearance ranked by the counts. 2) converts FASTA files into the Galaxy data file type “tabular” (tab-separated text files). 3) converts dashes in to tabs and 4) extracts selected columns of a given tabular file. 5) joins files by a selected identifier column. (PNG 4714 kb) [file 12864_2018_4954_MOESM2_ESM.png]

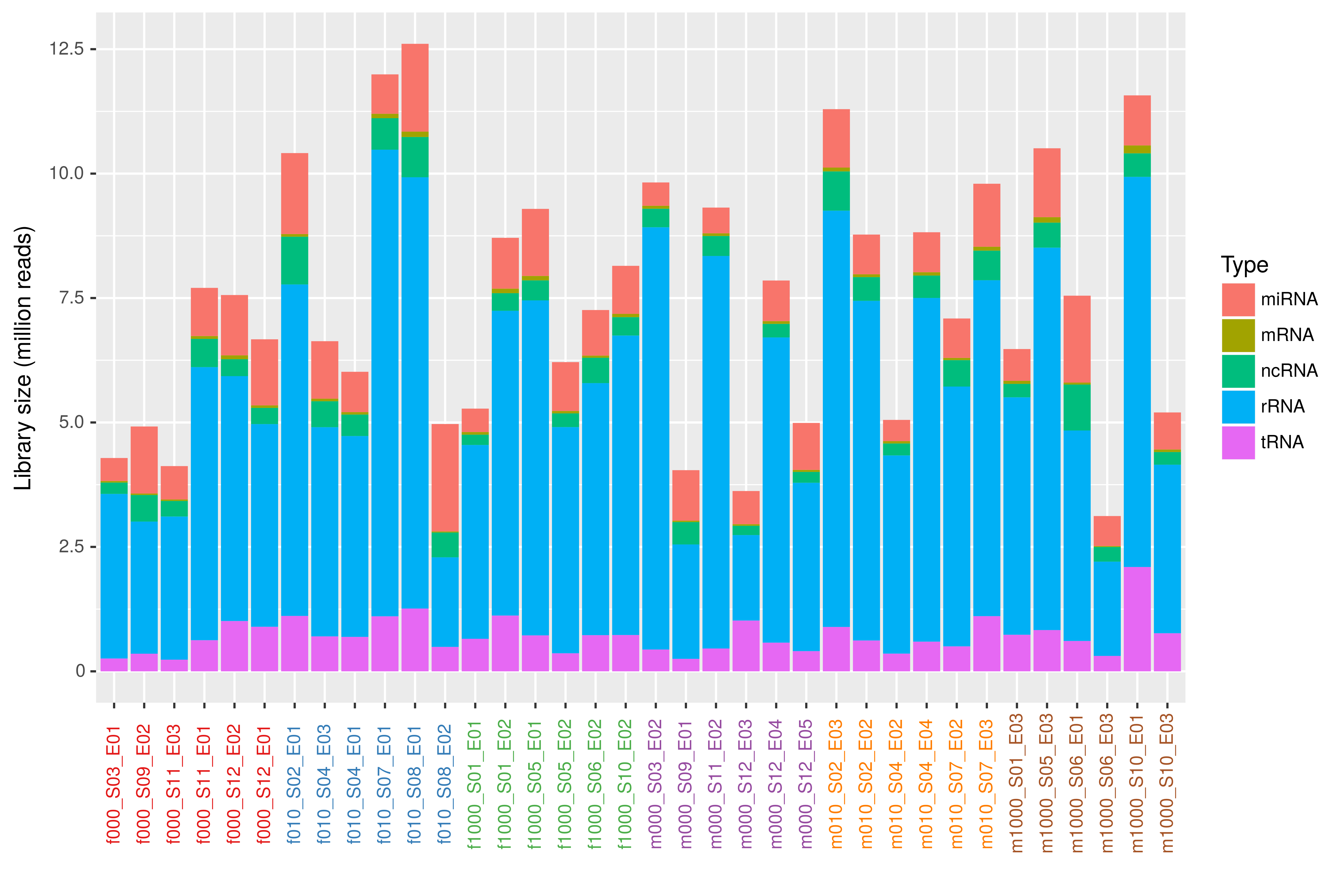

Supplement: Supplementary file 5 — Figure S3. Library sizes of mapped reads per RNA type. (PNG 617 kb) [file 12864_2018_4954_MOESM5_ESM.png]

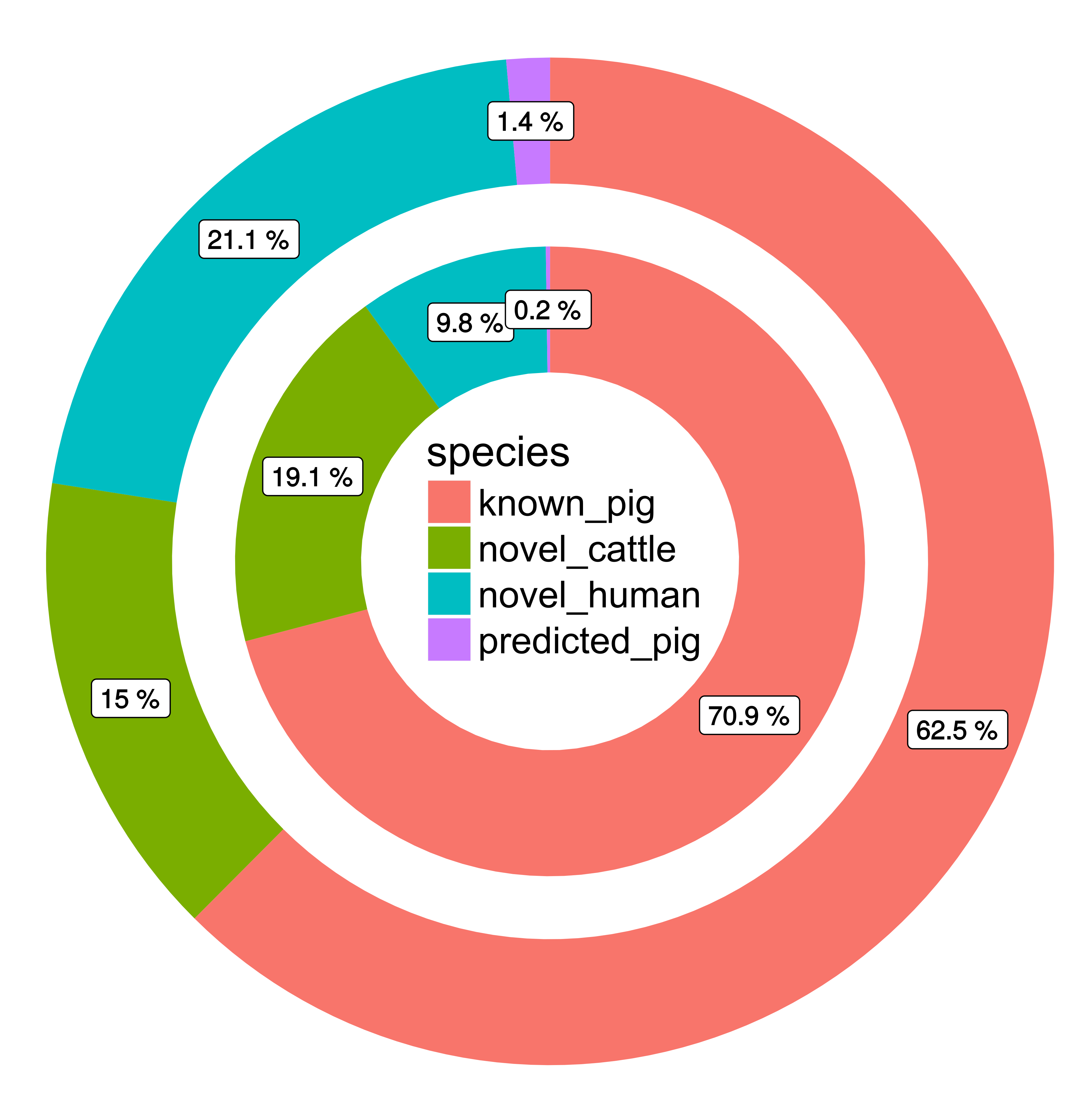

Supplement: Supplementary file 7 — Figure S4. Known and novel miRNAs and species annotation - unique sequences and read counts. In the outer ring the mapped unique sequences are shown related to mapped species. The inner cycle represents the corresponding read counts per species. (PNG 468 kb) [file 12864_2018_4954_MOESM7_ESM.png]

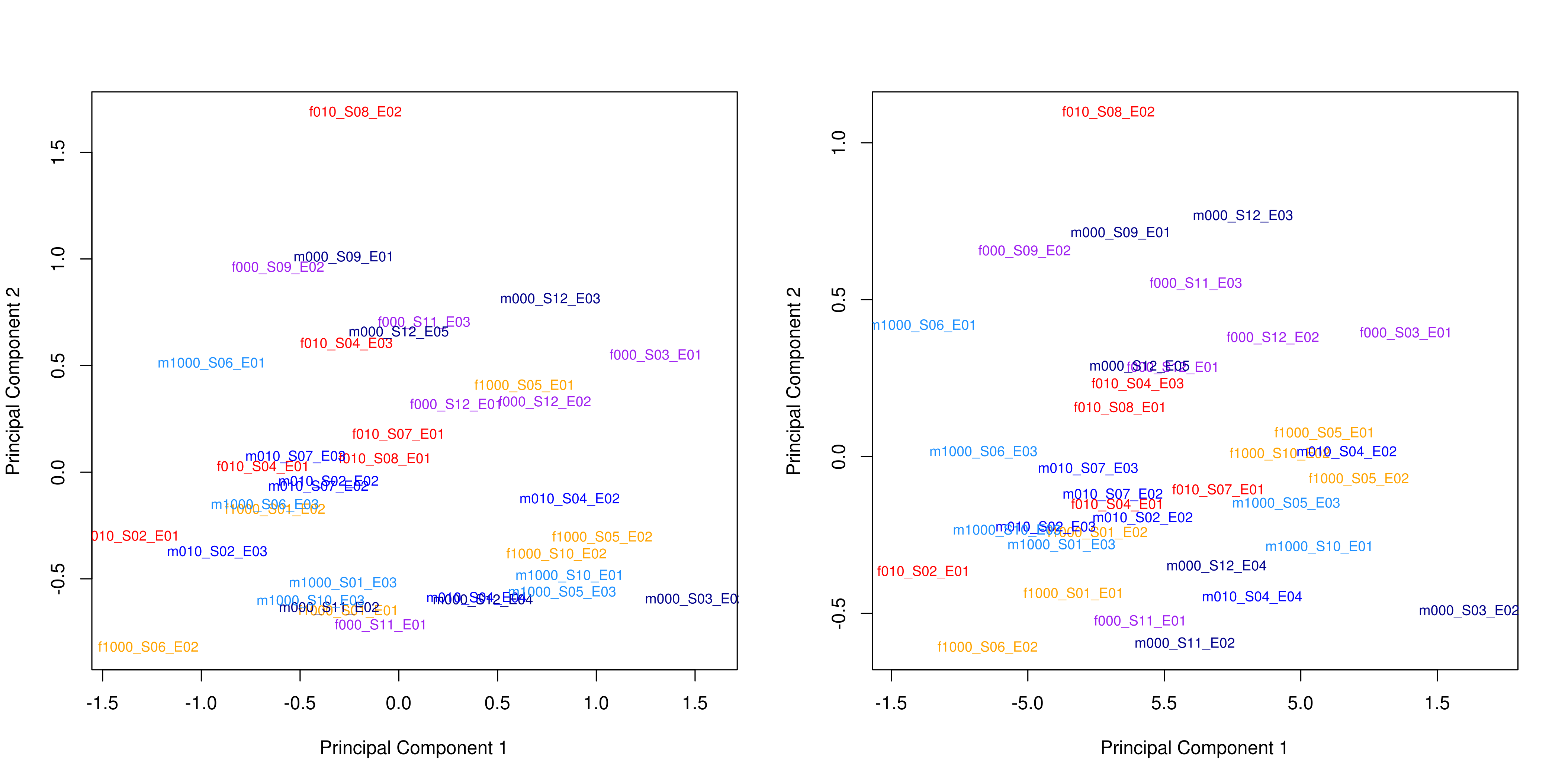

Supplement: Supplementary file 8 — Figure S5. MDS plots of the top 300 and 2000 isomiRs. The different colors represent the six different treatment groups (three different E2 doses with respective male and female embryos). (PNG 533 kb) [file 12864_2018_4954_MOESM8_ESM.png]

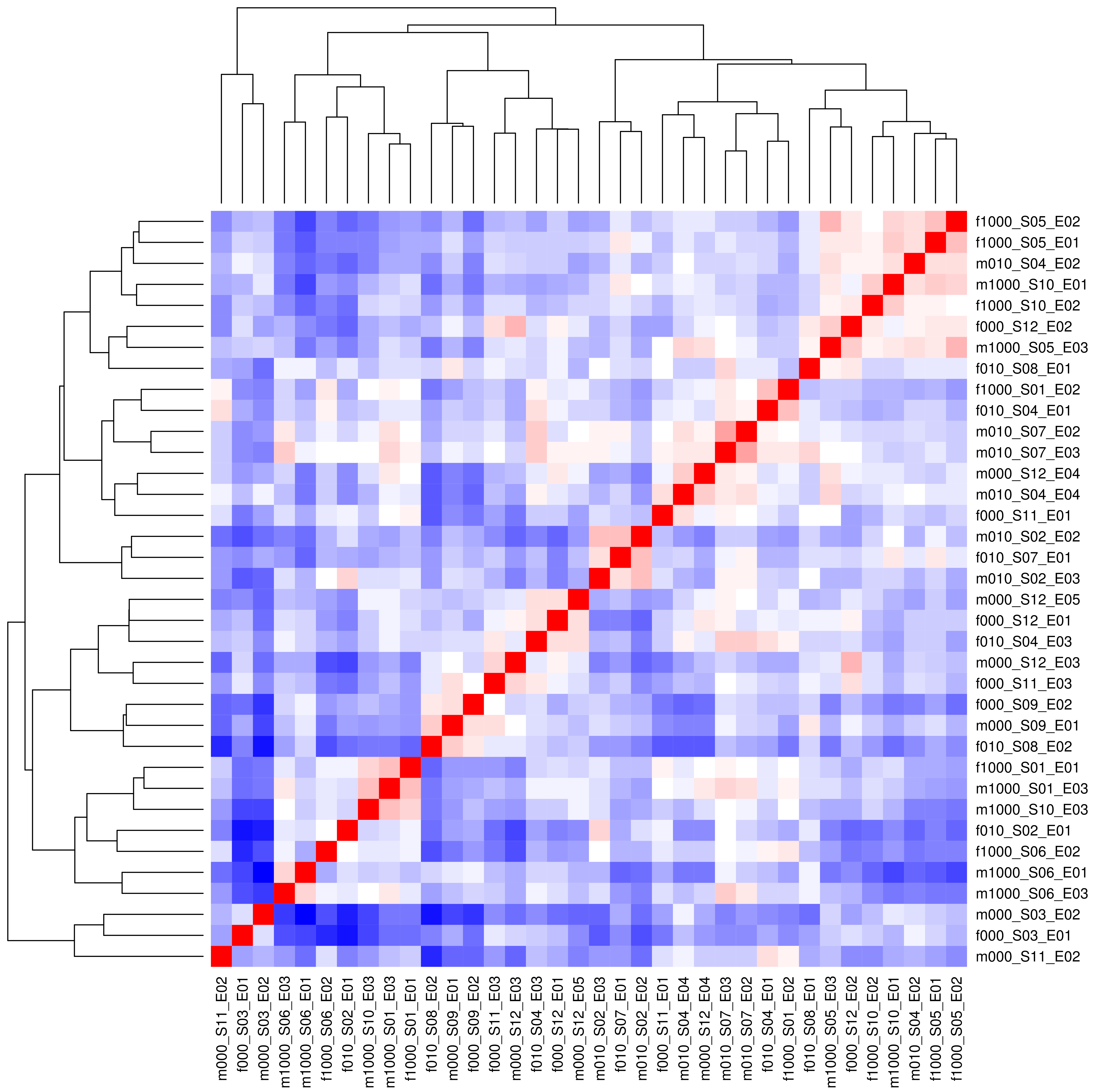

Supplement: Supplementary file 9 — Figure S6. Distance heatmap of all samples. (PNG 1065 kb) [file 12864_2018_4954_MOESM9_ESM.png]
